# MoSun4 Contributes to Pathogenicity and Is Associated with Altered Expression of Virulence-Related Genes in *Magnaporthe oryzae*

**DOI:** 10.3390/jof12080605

**Published:** 2026-08-13

**Authors:** Huimin Li, Zhenhe Su, Xiaomeng Liu, Lemeng Dong, Qinggang Guo

**Affiliations:** 1 Plant Protection Institute, Hebei Academy of Agriculture and Forestry Sciences, National Collection of Plant-Associated Microbes (Hebei), IPM Innovation Center of Hebei Province, International Science and Technology Joint Research Center on IPM of Hebei Province, Baoding 071000, China; 2 Plant Hormone Biology Group, Swammerdam Institute for Life Sciences, University of Amsterdam, Science Park 904, 1098 XH Amsterdam, The Netherlands

**Keywords:** *Magnaporthe oryzae*, pathogenicity, MoSun4, secreted protein, transcriptome

## Abstract

The SUN family protein MoSun4 in *Magnaporthe oryzae* has been previously implicated in mitophagy and has potential as a target for reducing rice blast, but its role as a secreted protein remains poorly understood. In this study, signal peptide prediction and yeast secretion assays confirmed MoSun4 signal peptide function, and co-localization revealed the localization of the extra-invasive hyphal membrane (EIHM)-associated apoplastic compartment or matrix, establishing MoSun4 as a secreted protein. In addition, deletion of the *MoSUN4* gene reduced the hyphal growth, conidiation and virulence of *M. oryzae*. We further showed that MoSun4 is involved in regulating the expression of virulence-related genes, including multiple genes involved in cell wall degradation (*eglC*, *eglD*), secondary metabolism (*gliK*), and melanin biosynthesis (*SDH1*, *BUF1*, *Cmr1*, *ALB1*). Collectively, our study reveals that MoSun4 is a secreted protein that contributes to pathogenicity and is associated with altered expression of virulence-related genes, providing new insights into the functions of SUN family proteins.

## 1. Introduction

Cereal species such as rice, wheat and maize are the staple food sources in many areas of the world. Diseases affecting cereal crops exert a particularly great economic and agronomic impact on global grain production and the grain industry. To develop effective means to control fungal diseases, several control measures against fungal diseases, including fungicides and resistant cultivars, have been developed [1,2]. However, due to the constant development of fungicide resistance, which is now of global concern, new targets for disease intervention are urgently required. Today, technical advances, such as host-induced gene silencing (HIGS) and screening small-molecule inhibition, provide effective approaches to designing inhibitors and developing resistant cultivars based on pathogen targets [3,4,5]. Further identification of specific virulence genes in plant pathogens might provide an effective strategy for controlling fungal diseases.

Rice blast disease, known as the cancer of rice, is caused by *Magnaporthe oryzae*, leading to a reduction in production of over 10% worldwide [6,7,8,9]. In recent years, significant progress has been achieved in developing ways to withstand rice blast caused by *M. oryzae* through the application of fungicides and planting resistant rice. However, resistant varieties are easily lost after continuous cultivation because of the adaptability and rapid variation in rice blast strains in the field, and fungicide resistance is rapidly developed by the fungus, reducing the control effect [1]. Therefore, the identification of new targets for disease intervention and the development of previously unused chemicals are urgently needed.

The SUN family proteins in *Saccharomyces cerevisiae*, encoded by the homologous genes *SIM1*, *UTH1*, *NCA3*, and *SUN4*, are involved in diverse cellular processes including DNA replication, lifespan regulation, mitochondrial biogenesis, cell septation, and cell wall morphogenesis [10,11,12]. Moreover, they have been reported to have multiple cellular locations, including the cell wall, the extracellular space, and the mitochondria [13]. Its *Botrytis cinerea* homolog BsSun1 has been reported to be a secreted protein with potentially hyper-O-glycosylated regions and is involved in maintaining the structure of the cell wall, the extracellular matrix and pathogenesis in *B. cinerea* [14]. Yu et al. revealed that UvSun1 is required for the growth, cell wall integrity and pathogenicity of *Ustilaginoidea virens* [15]. Furthermore, Gastebois et al. demonstrated that *Aspergillus fumigatus* AfSun1p and *Candida albicans* Sun41p can specifically hydrolyze straight chain β-(1,3)-glucan, and represent a new family of glucan hydrolases (GH132) [16]. Therefore, SUN family proteins may play various roles in different fungi. In contrast to the existence of four SUN family proteins in *S. cerevisiae*, there is only one SUN family protein homolog in *M. oryzae*, MoAti1, which is specifically required for mitophagy by facilitating the recruitment of MoAtg8 to mitochondria. Notably, no homologs of MoAti1 were found in rice and human protein databases, making it a potential candidate for drug targeting. Prior studies have further shown that HIGS targeting *MoATI1* effectively controls rice blasts, which suggests the potential feasibility of MoAti1 as a new target for novel anti-rice blast measures [17].

Based on this prior research, we identified the SUN family protein, MGG_00505 (previously reported as MoAti1 and referred to here as MoSun4 due to its SUN-domain annotation), and investigated its potential secreted function during host infection. We found that the signal peptide of MoSun4 is functional, and MoSun4 can be secreted into the extra-invasive hyphal membrane (EIHM)-associated apoplastic compartment or matrix. Furthermore, we revealed that MoSun4 is involved in regulating the pathogenicity, hyphal growth, and conidiation of *M. oryzae*. In addition, we showed that MoSun4 is involved in regulating the expression of virulence-related genes, including multiple genes involved in cell wall degradation (*eglC*, *eglD*), secondary metabolism (*gliK*), and melanin biosynthesis (*SDH1*, *BUF1*, *Cmr1*, *ALB1*). Taken together, our study reveals MoSun4 as a secreted protein that contributes to pathogenicity and is associated with altered expression of virulence-related genes, thereby broadening our understanding of SUN family functionality and highlighting its potential as a target for rice blast disease control.

## 2. Results

### 2.1. MoSun4 Is Secreted into the Extra-Invasive Hyphal Membrane

**Figure 1:**
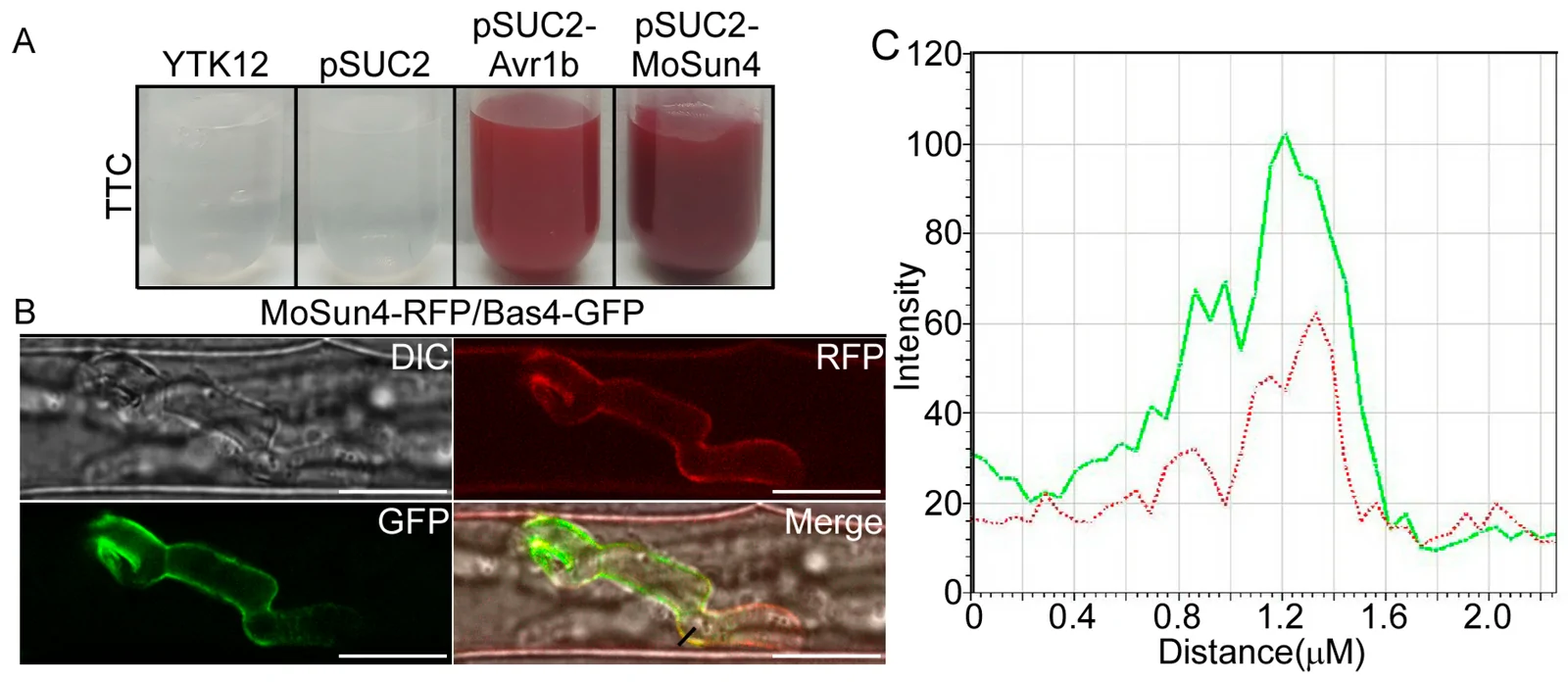
MoSun4 is a secreted protein. (**A**) The signal peptide function of MoSun4 was verified by a yeast signal peptide capture experiment. (**B**,**C**) The localization pattern of MoSun4 is consistent with a potential apoplastic effector role. MoSun4-RFP and Bas4-GFP vectors were co-expressed in the Guy11 strain to observe the subcellular localization of MoSun4 and Bas4 in plant cells. MoSun4-RFP was localized in the periphery of the infected mycelium and co-localized with Bas4-GFP after 24 h of infection. Solid black lines represent the labeled and merged signals. Bar = 10 μm. (**C**) Quantitative analysis of merged colocalization. Red, MoSun4-RFP; Green, Bas4-GFP.

MoSun4 has been reported to be partly located in the peripheral mitochondrial outer membrane in conidia and hyphae of *M. oryzae* [17]. However, its localization during the infection of rice has not been reported. Therefore, we performed signal peptide prediction using SignalP 5.0. The results show that the N-terminal of the MoSun4 protein has a typical secretion SP sequence (Figure S1). Subsequently, we validated the activity of the MoSun4 signal peptide sequence using a yeast secretion assay. The empty pSUC2 and Avr1b-SP vectors were used as negative and positive controls, respectively. The TTC results indicate that MoSun4-SP successfully secretes invertase (Figure 1A). Previous studies have indicated that the effectors are secreted and accumulated at the biotrophic interfacial complex (BIC) or within the extra-invasive hyphal membrane (EIHM). To study MoSun4 localization in the host, we expressed the *MoSUN4*:RFP fusion gene under the control of its native promoters in Guy11 and observed RFP fluorescence expression in the invaded rice sheath cells at 24 hpi using confocal fluorescence microscopy. Accordingly, fluorescence was co-localized with the well-established apoplastic effector Bas4 at the EIHM-associated apoplastic compartment or matrix, indicating that the localization pattern of MoSun4 is consistent with a potential apoplastic effector role (Figure 1B,C). When considered together, these data indicate that MoSun4 is a secreted protein.

### 2.2. MoSun4 Is Required for the Virulence of M. oryzae

**Figure 2:**
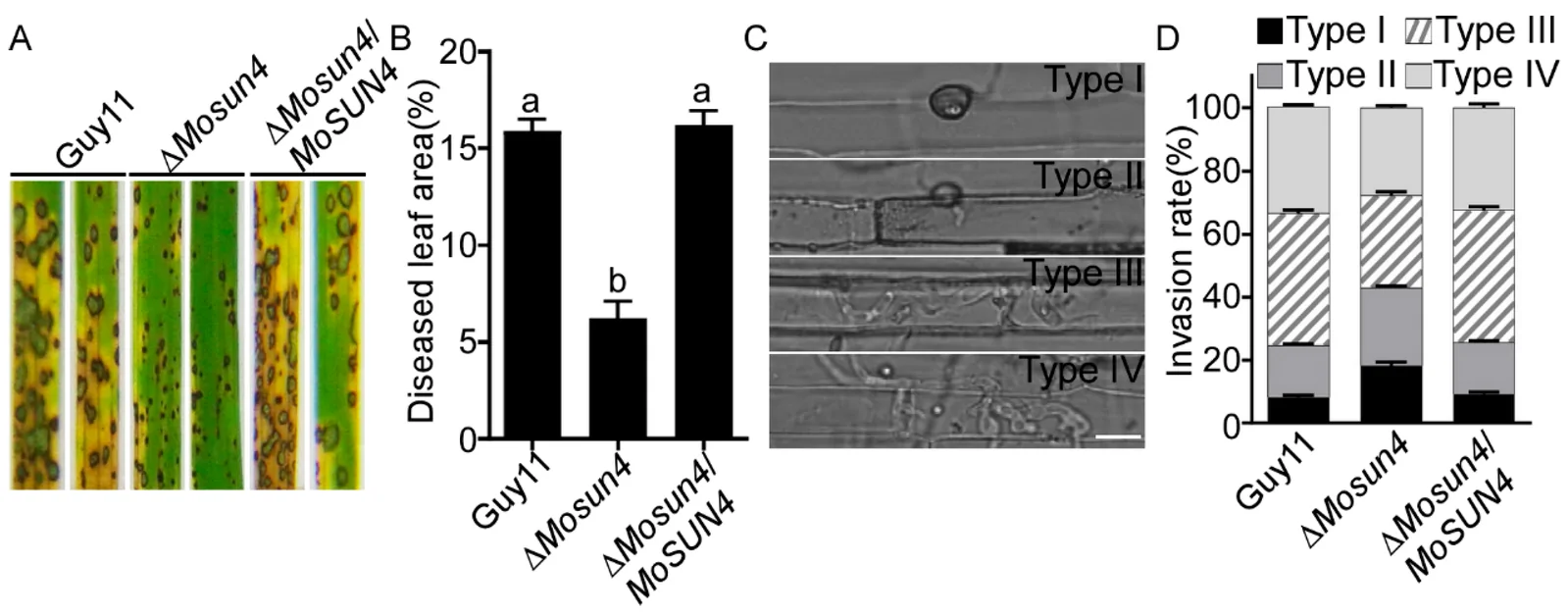
MoSun4 is required for the virulence of *M. oryzae*. (**A**) Pathogenicity assay. Four milliliters of conidial suspension (5 × 10^4^ spores ml^−1^) of each strain were used for spraying on Oryza sativa cv. CO39 and were photographed at 7 dpi. (**B**) Diseased leaf area analysis. Data are presented as a bar chart showing the percentage of lesion area analyzed by ImageJ 1.53t. Error bars represent the standard deviation (SD). Significant differences (*p* < 0.05) were determined by one-way ANOVA with Duncan’s post hoc test, as indicated by different letters. (**C**,**D**) Statistical analysis for each type of invasive hyphae (IH) shape. Type I, no penetration; type II, only a single IH without branches; type III, one to three branches but restricted in one cell; type IV, more than three branches and extended to neighboring plant cells. For each tested strain, 100 infecting hyphae (*n* = 100) were counted per replicate, and the experiment was repeated three times with similar results.

To examine the roles of MoSun4, we generated the Δ*Mosun4* mutant (Figure S1) and inoculated conidial suspensions of Guy11, Δ*Mosun4* and complementation strains on the susceptible rice cultivar CO39. The Δ*Mosun4* mutant was significantly reduced in virulence on rice (Figure 2A,B). Subsequently, we performed an infection assay on rice sheaths and observed IH growth at 100 appressorium penetration sites at 36 hpi. We rated the hyphal growth as type I, type II, type III, and type IV (Figure 2C,D). Guy11 and the complementation strains exhibited approximately 90% successful appressorium penetration events, with more than 70% type III and IV IH at 36 hpi (Figure 2D). In contrast, only 80% of Δ*Mosun4* appressoria had successful penetration, with less than 58% of penetration sites showing type III and IV IH growth (Figure 2D). The results showed that MoSun4 plays an important role in IH growth and host colonization, consistent with the results of a previous study [17].

### 2.3. MoSun4 Is Involved in the Mycelial Growth and Conidiation of M. oryzae

**Figure 3:**
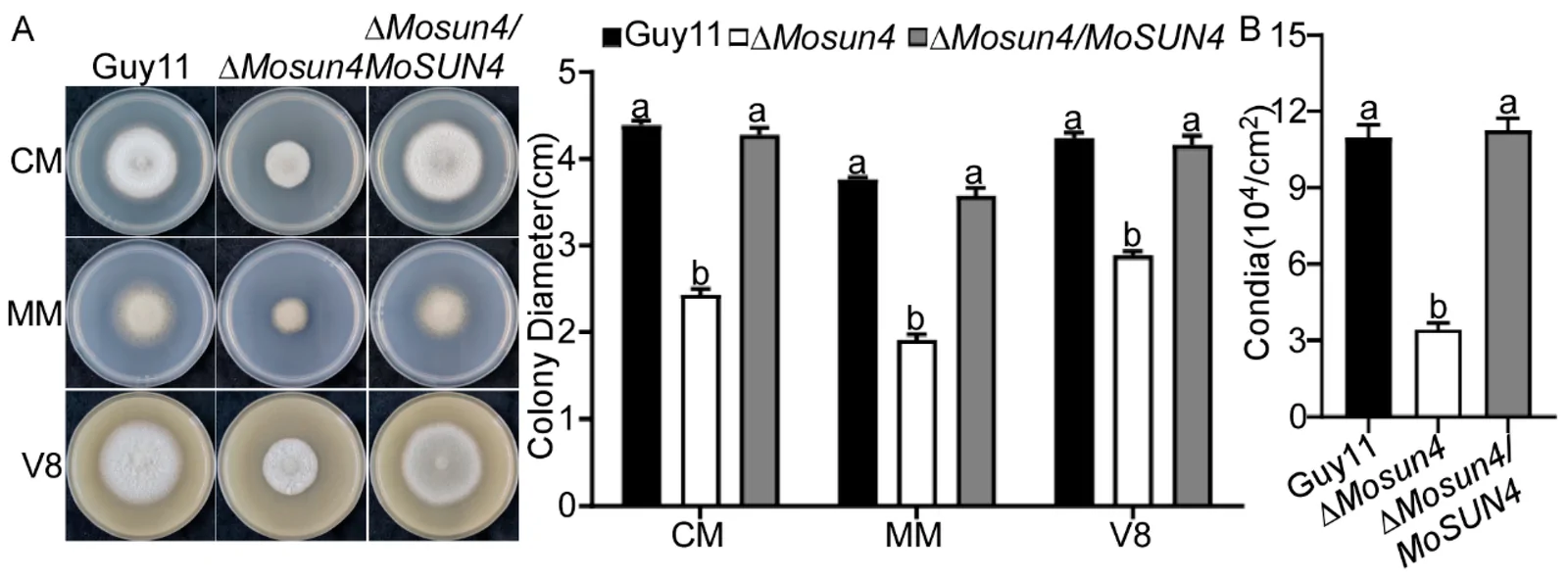
MoSun4 is involved in mycelial growth and conidiation. (**A**) Mycelial growth of Guy11, Δ*Mosun4*, and complementation strains grown on CM, MM, and V8. (**B**). Statistical analysis of conidia production for Guy11, Δ*Mosun4* and complementation strains. Error bars represent the standard deviation (SD). Significant differences (*p* < 0.05) were determined by one-way ANOVA with Duncan’s post hoc test, as indicated by different letters.

We further evaluated the growth of the Δ*Mosun4* mutant on complete (CM), minimal (MM), and V8 juice (V8) media. The Δ*Mosun4* mutant showed a slightly smaller colony diameter than Guy11 and Δ*Mosun4*/*MoSUN4* on all media types (Figure 3A). These results were further supported by statistical analysis (Figure 3A). As conidia play an important role during *M. oryzae* infection, we measured the conidial production of Guy11, the Δ*Mosun4* mutant, and Δ*Mosun4*/*MoSUN4* strains. We found that the Δ*Mosun4* mutant displayed apparent defects in conidiation in comparison to Guy11 and Δ*Mosun4*/*MoSUN4* strains (Figure 3B). These results indicate that MoSun4 is required for mycelial growth and conidiation.

### 2.4. MoSun4 Is Associated with Global Gene Expression in M. oryzae

**Figure 4:**
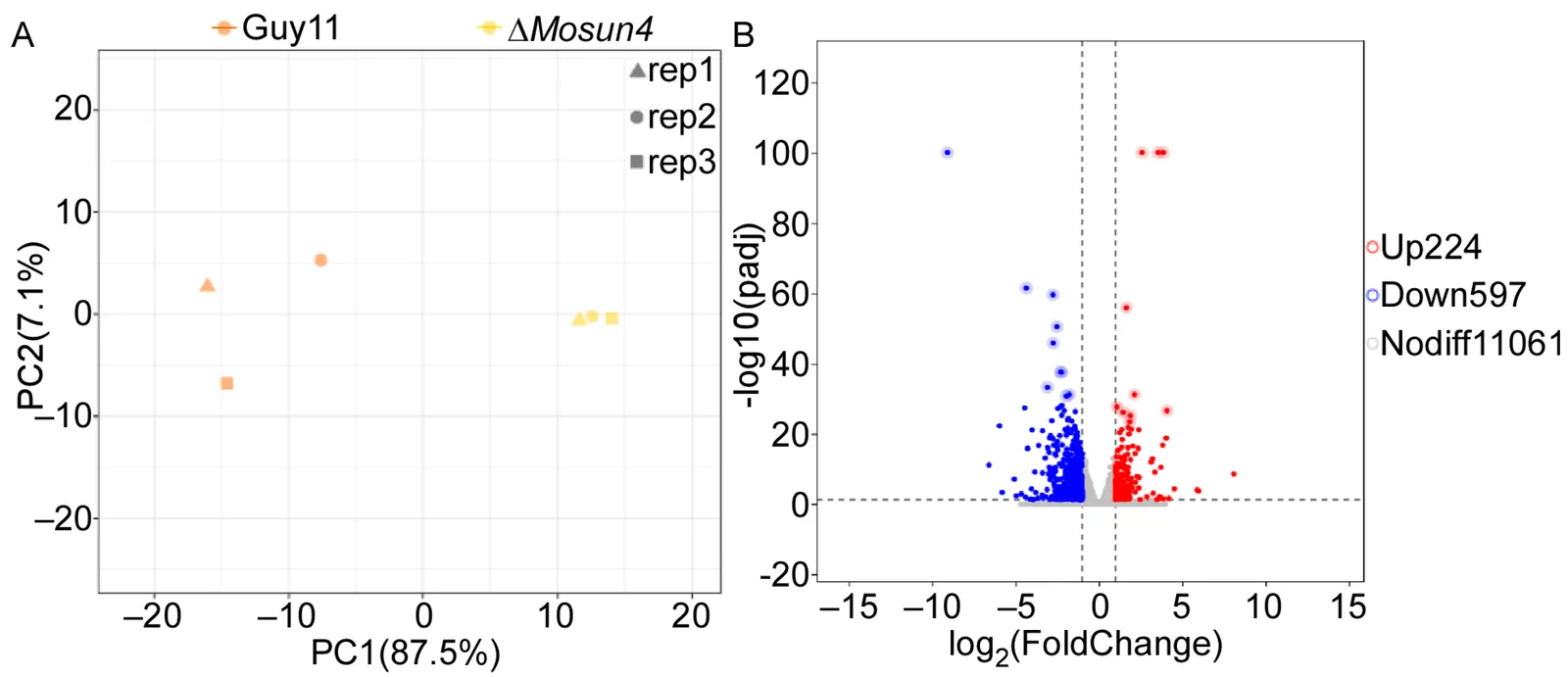
The PCA and volcano analyses of RNA-seq data from Guy11 and the Δ*Mosun4* mutant strains. (**A**) The PCA of RNA-seq data from Guy11 and the Δ*Mosun4* mutant strains. (**B**) The volcano of RNA-seq data from Guy11 and the Δ*Mosun4* mutant strains. The X and Y axes represent the logarithmic values of the fold change in gene expression and the padj, respectively. Each point corresponds to a differentially expressed gene. The red, blue, and gray dots represent genes with upregulated, downregulated, and non-significantly altered expression in the Δ*Mosun4* mutant, respectively.

**Figure 5:**
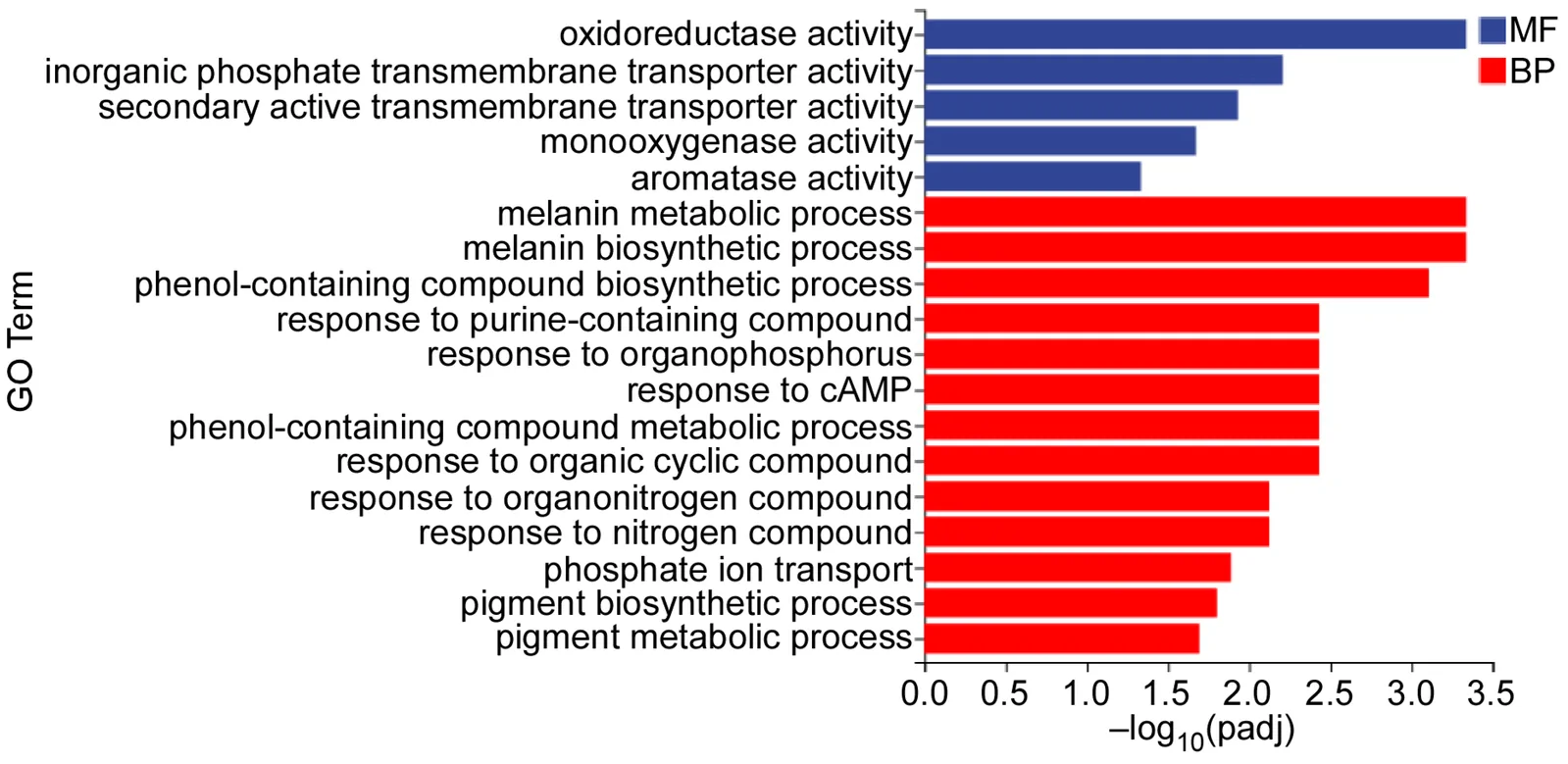
GO enrichment analysis of DEGs in the Δ*Mosun4* mutant. The length of each column represents the statistical significance of the genes enriched within the corresponding pathway. A pathway with a padj of less than 0.05 is considered to be significantly represented.

To examine the potential effect of MoSun4 on gene expression, we performed a transcriptome analysis involving Guy11 and the Δ*Mosun4* mutant. The sequencing filtering statistics of all samples are summarized in Table S1. Principal component analysis (PCA) revealed a distinct pattern of the global transcriptome between the two strains (Figure 4A). Additionally, differentially expressed genes (DEGs) were identified using the threshold |log_2_(Fold change)| > 1 and an adjusted *p*-value (padj) < 0.05, and DEGs analysis uncovered that 224 genes were significantly upregulated and 597 genes were significantly downregulated in the Δ*Mosun4* mutant (Figure 4B). To elucidate the functional categories enriched among transcriptional changes, GO annotation and enrichment analysis were conducted based on transcriptomic data. The GO enrichment analysis revealed significant enrichment of biological processes, molecular functions associated with melanin, phenol-containing compound metabolic and biosynthetic processes, and oxidoreductase and inorganic phosphate transmembrane transporter activity in the Δ*Mosun4* mutant (Figure 5). In addition, multiple response pathways to small signaling molecules, phosphate ion transport and pigment metabolism were also enriched (Figure 5). These results indicate that MoSun4 is essential for the regulation of redox metabolism, phosphate transport and melanin production.

### 2.5. MoSun4 Is Associated with Altered Expression of Virulence-Related Genes

**Figure 6:**
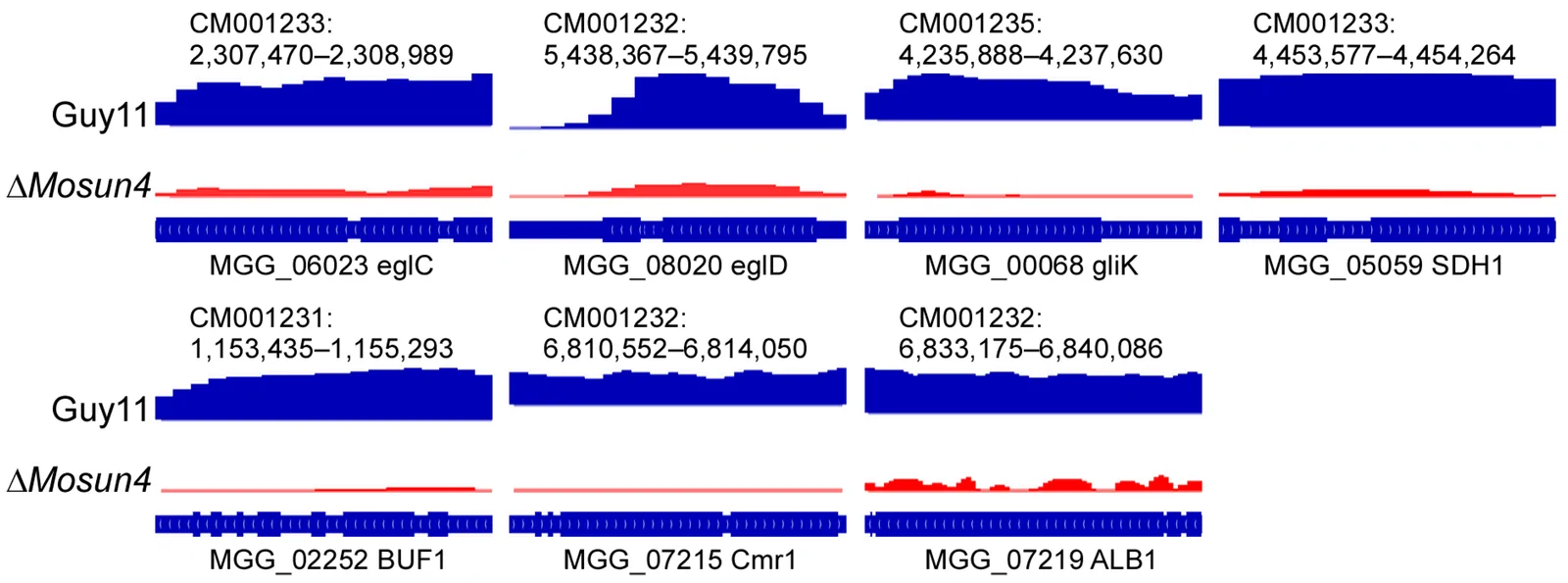
The relative gene expression levels are shown with screenshots of the integrative genomics viewer (IGV) display. Guy11, blue. Δ*Mosun4*, red.

**Figure 7:**
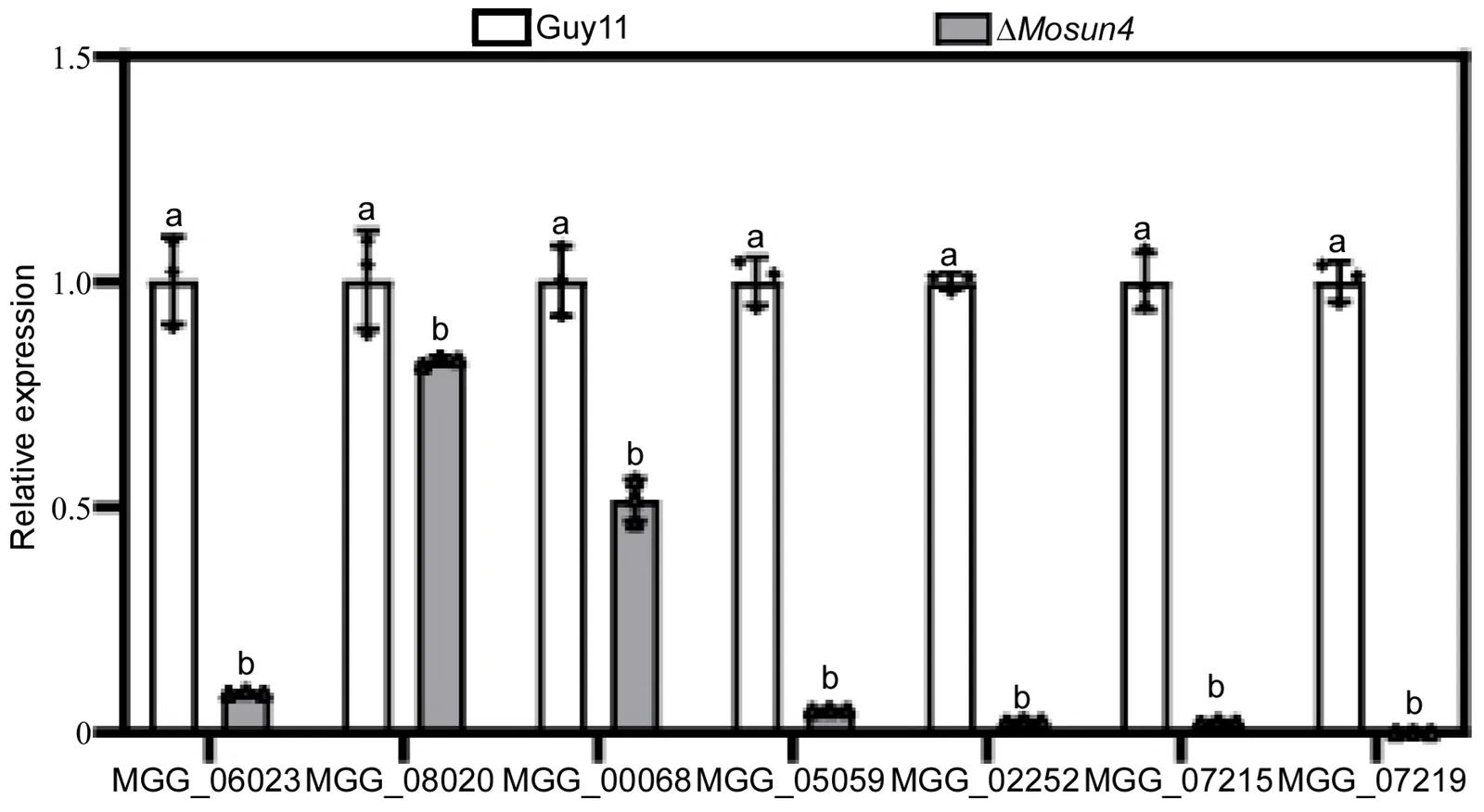
The relative expression of downregulated genes in the Δ*Mosun4* mutant. Error bars represent the standard deviation (SD). Significant differences (*p* < 0.05) were determined by one-way ANOVA with Duncan’s post hoc test, as indicated by different letters.

Given the significant enrichment of processes related to melanin biosynthesis and extracellular regions in the Δ*Mosun4* mutant, as determined by GO analysis, we further investigated whether MoSun4 is involved in regulating the expression of genes directly associated with melanin biosynthesis and pathogenicity. The integrative genomics viewer (IGV) display revealed drastically reduced transcript abundance of seven key genes, including multiple genes involved in cell wall degradation in the extracellular region (*eglC*, *eglD*), secondary metabolism (*gliK*), and melanin biosynthesis (*SDH1*, *BUF1*, *Cmr1*, *ALB1*) (Figure 6). Notably, *gliK* has been previously reported to play a role in regulating the pathogenicity of *M. oryzae* [18]. Consistent with the omics data, the relative expression of the seven genes mentioned above was higher in Guy11 than that in the Δ*Mosun4* mutant at the mycelium stages (Figure 7). These results indicate that MoSun4 is associated with altered expression of virulence-related genes.

## 3. Discussion

In this study, we characterized the biological function of MoSun4 (MGG_00505, formerly named MoAti1), a SUN family protein in *M. oryzae*. A key finding of this study is that MoSun4 is a secreted protein. Signal peptide prediction, yeast secretion assays, and confocal fluorescence microscopy confirmed that MoSun4 contains a functional signal peptide and is secreted to the EIHM-associated apoplastic compartment or matrix, where it co-localizes with the apoplastic effector Bas4 (Figure 1). Our results demonstrate that MoSun4 contributes to pathogenicity and is involved in regulating the expression of virulence-related genes, including those involved in melanin biosynthesis, cell wall degradation, and secondary metabolism (Figure 4 and Figure 5). These findings not only confirm the essential role of MoSun4 in fungal virulence and development (Figure 2 and Figure 3) but also reveal a novel transcription-centered regulatory mechanism, which complements and extends the recently reported function of MoSun4 as a mitochondrial mitophagy receptor [17]. Together, these studies establish MoSun4 as a dual-localized, multifunctional regulator critical for the infection cycle of *M. oryzae*.

While both our study and prior studies [17] confirm the indispensable role of MoSun4 in pathogenicity, they uncover distinct facets of its multifunctional nature. Shi et al. confirmed that MoSun4 participates in mitophagy by recruiting the core autophagy-related protein MoAtg8 to mitochondria through AIM/LIR motifs, and that MoSun4-mediated mitophagy is required for virulence in *M. oryzae* [17]. In contrast, our study identified MoSun4 as a secreted protein that modulates pathogenicity through transcriptional regulation of virulence-associated genes. Furthermore, in addition to its reported localization at the peripheral mitochondrial outer membrane, our work reveals an additional EIHM-associated apoplastic compartment or matrix localization for MoSun4, suggesting potential roles in directly or indirectly regulating plant immunity. These observations are not mutually exclusive. Specifically, MoSun4 may function in mitochondria to support mitophagy during early infection or under nutrient stress, while its secreted form at the EIHM may modulate host immunity or fungal cell wall dynamics during host colonization. However, the precise molecular switch governing these distinct localizations and functions, and whether they are functionally interconnected, remains an important but unresolved question. Such functional duality has been reported for other SUN family proteins [19]. MoSun4 dual roles may result from either proteolytic cleavage generating isoforms with differential mitochondrial targeting signals or signal peptide usage, or unconventional secretion bypassing the ER–Golgi pathway [20,21]. Whether the transcriptional changes observed in the Δ*Mosun4* mutant are a direct consequence of effector activity or an indirect effect of impaired mitophagy remains to be determined.

Beyond its effector and mitophagy-related regulatory roles, emerging evidence indicates that SUN family proteins are broadly involved in maintaining cell wall morphology and mediating stress responses [22,23,24]. In addition, a previous study has revealed that *Aspergillus fumigatus* AfSun1p and *Candida albicans* CaSun41p can specifically bind to and act on linear β-(1,3)-glucans [16]. This may suggest that in the cell wall, these SUN proteins act to provide building blocks to other enzymes that are necessary for cell wall biogenesis and/or to counteract the activity of cell wall degradation enzymes. The finding that Sun4 belongs to the GH132 glucan hydrolase family [16] raises the possibility that it may also have a direct biochemical function in the apoplast, such as modifying host cell walls or fungal cell walls during infection.

The attenuated virulence observed in the MoSUN4 deletion mutant is a comprehensive outcome derived from multiple physiological disorders. Impaired vegetative growth and decreased conidiation restrict the basic infectious source of *M. oryzae*. Meanwhile disrupted mitochondrial function and disturbed metabolic homeostasis further weaken the stress tolerance and invasive growth ability during host colonization. These multiple defects jointly lead to the compromised pathogenicity of the mutant strain. Notably, MoSun4 is a fungal-specific protein with no homologs in rice or human proteins, making it an ideal target for disease intervention, and HIGS targeting *MoSUN4* has been shown to significantly enhance rice resistance to blast without compromising agronomic traits [17]. Our transcriptome data and the validation results of qRT-PCR further support that MoSun4 regulates multiple fungal-specific virulence and metabolic pathways, strengthening its potential as a target for HIGS or small-molecule inhibitor development.

In conclusion, this study reveals that MoSun4 is a secreted protein that contributes to pathogenicity and is associated with altered expression of virulence-related genes. Our findings deepen understanding of the functions of SUN family proteins and secreted protein-mediated pathogenicity in fungi, providing a promising target for the sustainable control of rice blast. However, the molecular mechanisms underlying this process remain largely unknown. The RNA-seq data show broad transcriptional reprogramming in Δ*Mosun4*, but it is not yet clear whether these changes are directly driven by MoSun4 or arise indirectly from its loss, for example, through disrupted cellular processes or signaling pathways. We also have not identified the host targets or fungal partners that MoSun4 may interact with, nor have we characterized its predicted biochemical activity.

To address these questions, we propose biochemical assays to determine whether MoSun4 functions as a GH132 glucan hydrolase, along with mutagenesis of conserved catalytic residues to define the contributions of its putative active site. Furthermore, we have not evaluated the sensitivity of the Δ*Mosun4* mutant to cell-wall-perturbing reagents and antifungal compounds such as calcofluor white, micafungin and itraconazole. Whether MoSun4 participates in cell wall homeostasis and antifungal resistance remains to be determined in future work. The expression of MoSun4 in rice tissue should be tested to determine its capacity to modulate host immunity, while yeast two-hybrid and co-immunoprecipitation screens can seek interaction partners from both fungal and plant origins. Together, these approaches should help distinguish direct from indirect effects and clarify the molecular context in which MoSun4 operates.

## 4. Materials and Methods

### 4.1. Strains, Media and Growth Conditions

*Magnaporthe oryzae* wild-type strain Guy11 was used in this study and generously provided by Professor Zhengguang Zhang, College of Plant Protection, Nanjing Agricultural University. For mycelial growth, small agar blocks (2 mm × 2 mm) were excised from the edge of 4-day-old cultures and placed onto complete medium (CM), minimal medium (MM) or V8 juice medium (V8) for culture in the dark at 28 °C for 7 d. The growth diameter was measured after incubation for 7 d. Liquid CM was used to prepare mycelia for DNA and RNA extraction. For conidia production, mycelia were grown in the dark on V8 medium at 28 °C for 7 d, followed by constant illumination for 3 d under fluorescent light.

### 4.2. Targeted Gene Deletion

The Δ*Mosun4* mutant used in this study was generated using the one-step gene replacement strategy. Two fragments with 1.0 kb of sequences flanking the targeted gene were PCR-amplified with primer pairs. The resulting PCR products were ligated to the hygromycin resistance cassette (*HPH*) released from pCX62. The 3.4 kb fragment, which included the flanking sequences and the HPH cassette, was transformed into Guy11 protoplasts. Putative mutants were screened by PCR. A Fungal Genomic DNA Kit (DP317, Tiangen, Beijing, China) was used to extract genomic DNA from the mycelium of Guy11 and Δ*Mosun4* strains. The primers used in this study are listed in Table S5.

### 4.3. Complementation of MoSUN4-Knockout Mutants

The complement fragment, which contains a 1.5 kb native promoter region and the entire coding region of the *MoSUN4* gene, was amplified by PCR and inserted into the pYF11 vector (bleomycin resistance) carrying the green fluorescent protein (GFP) using the yeast gap-repair approach [25]. The resulting construct was then used to complement the Δ*Mosun4* mutant via protoplast transformation. The *MoSUN4* complement transformants were picked with toothpicks and placed into CM Petri dishes. The mycelium of the transformants was picked onto a slide and the green fluorescence was observed under a Leica DM6B fluorescence microscope (Leica Microsystems CMS GmbH, Mannheim, Germany).

### 4.4. Construction of the MoSun4-RFP Plasmid Vector

The construction fragment, which contains a 1.5 kb native promoter region and the entire coding region of the *MoSUN4* gene, with an RFP fragment fused to the C-terminus of MoSUN4, was inserted into the modified no-GFP pYF11 vector (G418 resistance) using the yeast gap-repair approach [25].

### 4.5. Virulence and Invasive Growth Assays

For infection, conidia were harvested from 10-day-old V8 agar cultures, filtered, and resuspended to a concentration of 5 × 10^4^ spores ml^−1^ in a 0.2% (*w*/*v*) gelatin solution. The conidia were sprayed onto 2-week-old seedlings of rice (*Oryza sativa* cv. CO39). In addition, 5 mL of a conidial suspension of each treatment was sprayed onto rice leaves. For the detached barley inoculation assay, conidia were suspended to a concentration of 1 × 10^5^ spores ml^−1^ in a 0.2% (*w*/*v*) gelatin solution. Inoculated plants were kept in a growth chamber at 25 °C with 90% humidity and in the dark for the first 24 h, followed by a 12 h/12 h light/dark cycle. Lesion formation was observed daily and recorded by photography at 7 dpi. The degree of disease lesions was analyzed by ImageJ 1.53t [26].

For observation of penetration and invasive growth in rice cells, conidial suspensions (1 × 10^5^ spores ml^−1^) of Guy11, Δ*Mosun4* and Δ*Mosun4*/*MoSUN4* strains were injected into rice leaf sheaths. The inner epidermis of infected sheaths was harvested at various hours post-inoculation by Guy11 and mutant strains and observed under a Leica DM68 microscope (Leica Microsystems CMS GmbH, Mannheim, Germany).

### 4.6. Yeast Signal-Sequence (SP) Trap System

We performed signal peptide prediction of the *MoSUN4* gene using SignalP 5.0 [27]. The yeast SP secretion experiment was performed to verify the function of the predicted SP of MoSun4, as previously described [28]. The predicted SP sequences of target genes were cloned into the pSUC2 vector. An empty pSUC2 vector and a vector containing the Avr1b SP served as negative and positive controls, respectively. The recombinant plasmids were transformed into *Saccharomyces cerevisiae* YTK12. Positive clones were screened on the CMD/-W medium containing 0.67% yeast nitrogen base without amino acids, 0.075% W dropout supplement, 2% sucrose, 0.1% glucose, and 2% agar. Positive colonies were transferred to the YPRAA medium plates (2% peptone, 1% yeast extract, 2% raffinose, and 2 mg/mL antimycin) for invertase secretion. Moreover, invertase enzymatic activity was detected by the reduction of 2,3,5-triphenyltetrazolium chloride (TTC) to insoluble red-colored 1,3,5-triphenylformazan according to procedures and conditions described previously.

### 4.7. Epifluorescence Microscopy Observation

Invasive hyphae expressing fluorescent protein-fused target genes were incubated under appropriate conditions. The constructs carrying MoSun4-RFP and Bas4-GFP were transformed into the wild-type Guy11 strain. For microscopic observation of penetration and hyphal expansion in rice tissue, conidial suspensions (1.5 × 10^5^ spores ml^−1^) were injected into rice sheaths with a 1 mL syringe. After 24 h of incubation at 28 °C, the inner epidermises of infected sheaths were observed under a Leica TCSSP8 (100 × oil) microscope (Leica Microsystems CMS GmbH, Mannheim, Germany).

### 4.8. RNA-Seq Analysis

Total RNA was extracted from *axenic mycelia* of Guy11 and the Δ*MoSun4* mutant strains using Invitrogen TRIzol reagent (Thermo Fisher Scientific, Inc., Waltham, MA, USA) with three biological replicates per group. The RNA quality and quantity were determined using an Agilent 2100 Bioanalyzer instrument (Agilent Technologies, Inc., Santa Clara, CA, USA). Subsequently, mRNA libraries were generated using a NEBNext Ultra II RNA Library Prep Kit for Illumina (New England Biolabs Inc; Ipswich, MA, USA) and high-throughput RNA sequencing studies were performed using an Illumina NovaSeq Xplus (Illumina, Inc., San Diego, CA, USA). The filtered reads were mapped to the reference genome of *M. oryzae* strain 70-15. The HISAT2 (v2.1.0) software is used to construct an index of the reference genome, and the paired-end clean reads are subsequently aligned to the reference genome using HISAT2. The read counts were generated using HTSeq (v0.9.1), and the FPKM (Fragments Per Kilo bases per Million fragments) was calculated. Differentially expressed genes (DEGs) were defined as genes with |log2(Fold change)| > 1 and an adjusted *p* value (padj) < 0.05 (Benjamini–Hochberg), as identified by DESeq2 (v1.38.3). PCA was performed using the R package PCAtools (v2.18.0). Based on DEG data, the volcano plot was generated using R package ggplot2 (v3.5.0), and GO enrichment analysis was performed through the R package clusterProfiler (v4.10.1). The RNA-seq and bioinformatics services were provided by Personal Biotechnology Co., Ltd. (PersonalBio, Shanghai, China).

### 4.9. RNA Extraction and qRT-PCR Analysis

Total RNA of 2-day-old *M. oryzae axenic mycelia* was isolated using a FastPure Universal Plant Total RNA Isolation Kit (RC411, Vazyme, Nanjing, China) according to the manufacturer’s manual. RNA quantity and quality was measured using Nanodrop ND-1000 and 1% agarose gel electrophoresis. First-strand cDNA was synthesized from 1 μg total RNA using HiScript III RT SuperMix (R323, Vazyme, China). ChamQ Universal SYBR qPCR Master Mix (Q711, Vazyme, China) was used for quantitative RT-PCR (qRT-PCR) reactions. QRT-PCR was performed using the QuantStudio 3 Real-Time PCR System (Thermo Fisher Scientific, Inc., Waltham, MA, USA). The expression of each gene was normalized to the expression of internal control ACTIN, and the primers used for qRT-PCR are listed in Table S5. The relative expression level was calculated using the 2^−ΔΔCt^ method [28]. Each qRT-PCR reaction contained three technical replicates together with three independent biological replicates.

### 4.10. Statistical Analysis

All data represent the mean ± standard deviation (SD) of at least three replicates. Statistical analysis was conducted using one-way analysis of variance (ANOVA) with Duncan’s new multiple range test, carried out via SPSS 2.0. The details of statistical tests are provided in the figure legends.

## Data Availability

The RNA-seq has been deposited in the Gene Expression Omnibus (accession number GSE330356). The original contributions presented in the study are included in the article/Supplementary Materials, further inquiries can be directed to the corresponding author.

## Supplementary Materials

The following supporting information can be downloaded at: https://www.mdpi.com/article/10.3390/jof12080605/s1, Figure S1: Signal peptide prediction of MoSun4 using SignalP 5.0; Figure S2: Diagram of *HPH* replacement in the coding region of *MoSUN4* and the PCR analysis of target deletion of the *MoSUN4* gene; Table S1: Filtering statistics of RNA-seq; Table S2: Mapping rate; Table S3: DEGs; Table S4: GO enrichment; Table S5: Primers used in this study; Table S6: KEGG enrichment.
