# Supplementary material for: MoSun4 Contributes to Pathogenicity and Is Associated with Altered Expression of Virulence-Related Genes in Magnaporthe oryzae
Source: J Fungi (Basel). 2026 Aug 13;12(8):605. doi: 10.3390/jof12080605 (PMC13514613; doi:10.3390/jof12080605)
Supplement: Supplementary file 1 [file jof-12-00605-s001.zip › Supplementary figures.pdf]

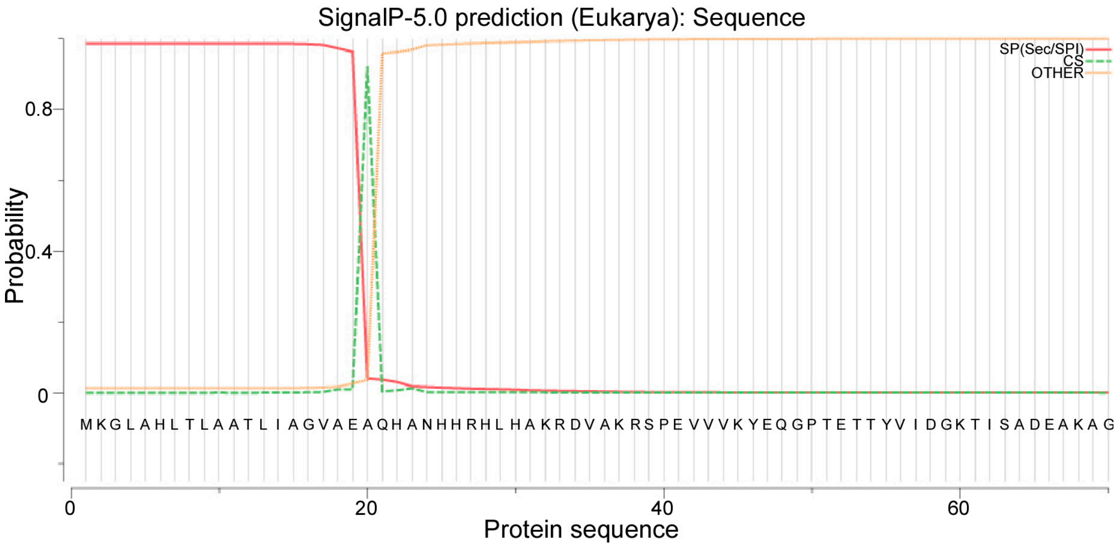

Figure S1. Signal peptide prediction of MoSun4 using SignalP 5.0.

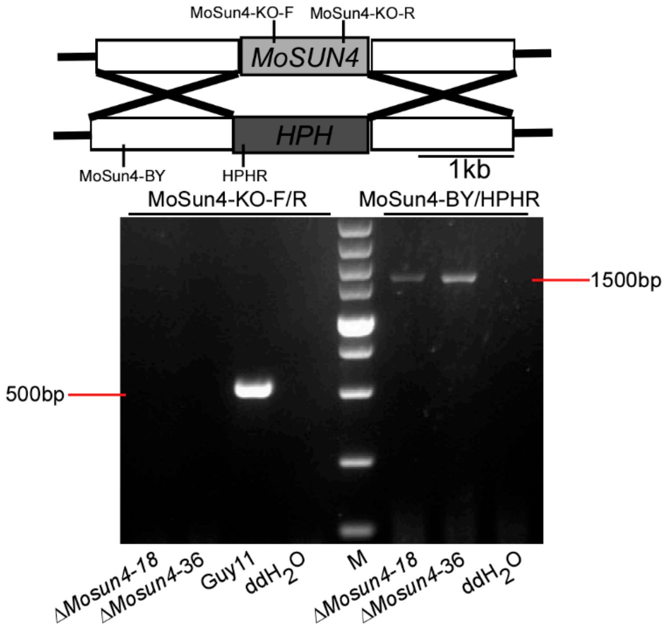

Figure S2. Diagram of *HPH* replacement in the coding region of *MoSUN4* and the PCR analysis of target deletion of the *MoSUN4* gene. PCR results with primers labeled as shown in the figure. M represents (DL5000 molecular marker). The lanes were the PCR results using DNA of labeled corresponding strains in the figure as template.
